# Imperative Surgery

**Published:** 1900-07

**Authors:** 


IMPERATIVE SURGERY. For the General Practitioner, the Specialist and the Recent Graduate. By Howard Lilienthal, M. D., Attending Surgeon to the Mt. Sinai Hospital, New York City. With numerous original illustrations from photographs and drawings. Pages 412. Price, $4, net. New York: The McMillan Company. 1900.

This work could have been called emergency surgery, and would have been about as appropriately named. It deals with emergencies such as come up suddenly and often require great presence of mind and originality, not to speak of skill. The book is needed by a large class and will no doubt have a large sale.

The introduction is devoted to instruments, surgical dressings, and their preparation, etc. Then the treatment of wounds, under all circumstances, operations in dwelling houses, and the various sources of infection are considered at some length. The consideration of regional surgery is given considerable space, covering the entire body, including the neck, extremities, thorax, abdomen, intestinal obstruction, and appendicitis. The last general division is devoted to suppuration of the liver and gall-bladder, strangulated hernia, peritonitis, rectum and anus, male and female genital organs, urinary system, eye and orbital region and the ear and mastoid region.

